# Supplementary material for: Bycatch survival of shortfin mako sharks (Isurus oxyrinchus) in the U.S. Atlantic pelagic longline fishery
Source: PLoS One. 2025 Sep 2;20(9):e0330999. doi: 10.1371/journal.pone.0330999 (PMC12404435; doi:10.1371/journal.pone.0330999)
Supplement: Attachment — Submitted filename: Review_POne.pdf [file pone.0330999.s003.pdf]

Review: Bycatch survival of shortfin mako sharks (*Isurus oxyrinchus*) in the U.S. Atlantic pelagic longline fishery

This paper presents a comprehensive assessment of AVS and PRS for shortfin mako shark from US fleets in the North Atlantic. It is well-written and comprehensive, and I particularly liked the approach of using MC simulation to produce overall bycatch mortality rates. I have no concerns about research ethics. There are two changes that I think are required before the paper is suitable for publication: (1) further detail and potential changes to the analyses in relation to hook depth information, and (2) consideration of bycatch rates predicted from only the significant predictors in the GAMM.

General Comments:

One idea that the authors could add to the Introduction is that the landings ban for mako in the North Atlantic was also intended to change fishing behaviour. The reasoning was that mako captures would cause losses (time, gear, etc.) and no monetary gain, providing an incentive to avoid interactions in the first place.

When quantifying the differences in fishing characteristics between the GOM and WNA, I was particularly interested in the information on hook depth. Where does this information come from? The discussion implies that hook depth information comes from POP observers, so is it possible that the relatively small differences (10s of m) reflect different estimation ability of observers in the two regions? Line 245 suggests that at least one longline set had hook depths of 2m (the lower boundary of the range), which seems improbable given the catenary curve of a mainline between floats combined with gangion lengths. Table 2 lists 'approximate' hook depths, yet values are presented as known elsewhere in the MS, so does this mean there are two sources? Throughout the MS, please identify and discuss how hook depth was determined and (more importantly) quantify underlying variability if the values were estimated. This would help the reader to assess if the weak relationships with hook depth from the regression analyses are meaningful, particularly given that they take different shapes among regions.

From my understanding of the MC simulation, the predicted relationships for all variables were sampled, even those that were non-significant and/or had very little evidence of a relationship. I am concerned that this approach exaggerates the differences in bycatch survival among the two regions. I would like to see an analysis using only the significant predictors from each region in the MC simulation and present the analysis now in the MS as a sensitivity analysis. If the authors have supporting literature demonstrating that their current approach is more statistically rigorous, this should be added as justification.

Specific Comments:

Line 66: Potential addition: '….. and that overfishing is occurring with high probability [8]'.

Line 71: perhaps 'fully implemented' is better terminology than 'adopted', given that there is always a time lag between when ICCAT recommendations are adopted and when they are fully in force in various jurisdictions.

Line 145-150: It would be worth mentioning why the modeling was split into two components, first a GLMM and then region-specific GAMMs. Presumably because this allowed for different relationships with predictor variables among regions?

Line 145-150: Were any interactions tested in the suite of models? After reading the results, I was interested if there was any evidence for an interaction between SST and soak time for WNA. This would also bolster the information in the discussion (lines 402-416).

Line 160: Is 0.7 a common threshold for collinearity? Please provide a supporting reference.

Line 187: Does the US fleet use wire leaders with weights or monofilament?

Line 217: Section on Bycatch survival. It would be helpful to a reader to have a specific statement on why the simulation was done. This would highlight how your analysis better captures uncertainty in both estimated rates when generating the overall value, as compared to other analyses that merely multiply the individual means.

Line 251: Were records considered incomplete if a single covariate was missing?

Line 265: '…. holding other variables constant at their predicted mean, …..' Similar comment for Line 302.

Line 353: consider replacing '=' with 'of'.

Lines 355-357 Re: bycatch survival. It would help the reader to have a simple statement that these are overall estimates (AVS*PRS).

Line 418: Figure 1 doesn't show the depth ranges that sharks were hooked at in the NWA or GOM. Maybe reference Figure 2?

Line 417-418: For an animal that regularly dives from the surface to > 400 m in a day (Figure 5), I have a hard time with the idea that the difference in mean hook depth between the two regions is biologically meaningful. Typically shallow-set longline is differentiated from deep-set longline by 100s not 10s of meters, with the associated expectation of differences in AVS and PRS.

Line 536-537: Please reword to '…. as possible are associated with very high survival probabilities.' Without assessing covariate effects, it is not possible to say that these actions maximize survival probabilities.

Figure 2: There seem to be no values on the x axes on each panel?

Supplementary File S2. Has the data been cleaned to only represent diving behaviour? Or are there still the portions of the tracks that represent times when the tag was at constant depth and/or drifting at the surface? Please clarify.
