# Supplementary material for: Bycatch survival of shortfin mako sharks (Isurus oxyrinchus) in the U.S. Atlantic pelagic longline fishery
Source: PLoS One. 2025 Sep 2;20(9):e0330999. doi: 10.1371/journal.pone.0330999 (PMC12404435; doi:10.1371/journal.pone.0330999)
Supplement: Attachment — Submitted filename: Response to Reviewers.docx [file pone.0330999.s004.docx]

Dr. Coelho,

Thank you for the opportunity to revise our manuscript and apologies for the delayed revision time. We have addressed the comments of the reviewers, and we believe our manuscript is improved as a result. Our responses to specific comments for each reviewer are available below, however we would first like to highlight some of the more general changes. All line numbers reference the line numbers in the document with the track-changes when all mark up in shown in balloons, where revisions are easier to see, unless otherwise noted.

The biggest change is that addressing the concerns of Reviewer 2 led us to completely revise the at-vessel survival analysis. Rather than run 2 separate GAMMs for different regions we used GLMMs and used data from all observer regions, and did not combine 3 regions into a single region (WNA) as we did originally. Our revised approach was as follows:

First, to test for regional differences in AVS we used a GLMM incorporating data from the 6 observer regions with > 100 mako shark observations (NED, NEC, MAB, SAB, FEC, GOM), with region as a categorical fixed effect. This is described in the methods on lines 393-404. We found evidence that AVS was greater in the 3 northern regions (NED, NEC, MAB) compared to the 3 southern regions (SAB, FEC, GOM). Which is similar to our result in the original manuscript, it encompasses more area and allows for more fine scale spatial variability to be observed. The results are presented in Table 2, and in the text on lines 651-654.

Next, to address the possibility of sex-biased survival raised by reviewer 2, before our analysis aimed at determining covariate effects on AVS, we used a GLMM that incorporated all available observations in which the sex of the shark was recorded. We use sex as a categorical fixed effect and found no evidence to suggest a difference in survival between sexes. Given this information, we felt justified in not including sex in our analysis to determine which covariates influenced AVS. This was advantageous because given the large proportion of sharks with no sex data recorded (~23%), including sex in further analyses would have led to a considerable loss of sample size and statistical power. The sex analysis is described in the methods on lines 411-418. The results are presented in Table 3 and on lines 654-656.

Finally, we pooled all observations for the analysis of covariate effects on AVS. We used logistic GLMMs for this analysis instead of a GAMM, as Reviewer 2 suggested. An advantage of this approach relative to our original analyses was that we could construct candidate models made up of different combinations of covariates and rank their support using AIC. The most supported model included only informative (significant) variables, addressing concerns of reviewers 1 and 2 in regards to making predictions from models with uninformative parameters. We found significant negative effects of soak time, SST, mainline length, and shark size on AVS, although the effects of soak time and SST were considerably stronger than mainline or shark size. The AUC for the top selected model was actually a bit higher than the AUC values for the two models in the original manuscript (although still not amazing). The new methodology is described in lines 410 – 473. Results are presented in Table 4 (AIC model selection), Figure 3 (beta coefficients of top model), Figure 4 (predicted effects of covariates in the top model), and in the text on lines 656-663, lines 782-789.

We altered the discussion of AVS as necessary to fit the results of the revised analysis, although the required changes were not extreme as the main effects of soak time and water temperature were also important in the new analysis.

When estimating bycatch survival using our Monte Carlo simulations, the biggest change is that instead of estimating bycatch survival for two regions (WNA and GOM), we estimated bycatch survival individually for the 6 regions with > 100 mako shark observations (lines 548-552). The results largely correspond with our previous findings (greater survival in northern regions compared to the GOM), but with more spatial detail. See Fig. 6 and lines 919-924.

We feel the revised analyses satisfies reviewer concerns while providing more detailed information on survival through the inclusion of data from a larger portion of the Atlantic than the original analysis. Still, the major findings of the original manuscript remain – PRS was greater than AVS, and survival varied regionally. Thus, our overall conclusions are the same.

To address concerns voiced to some degree by all reviewers, we added information on target species and more detail on differences in fishing and environmental conditions between regions. This includes a new Table 2, which shows the proportion of observed sets in each region targeting different species, the hook depth ranges associated with each target species, and the proportion of observed mako shark captures associated with targets in each region. A new Figure 2 shows distributions of covariates of interest between the 6 major observed regions in the form of a series of box plots. Also see lines 597-605, and lines 627-641 in the results.

In our previous manuscript we included distance between gangions in the analysis of AVS. We removed that from consideration in the revised analysis. There was no evidence it had any effect and there was little biological rationale for including it.

The abstract was revised as required to correspond to other revisions.

Our responses to individual comments are below

----------------------------------------------------------------------------------------------------------------

**Reviewer #1: Review: Bycatch survival of shortfin mako sharks (Isurus oxyrinchus) in the U.S. Atlantic pelagic longline fishery
This paper presents a comprehensive assessment of AVS and PRS for shortfin mako shark from US fleets in the North Atlantic. It is well-written and comprehensive, and I particularly liked the approach of using MC simulation to produce overall bycatch mortality rates. I have no concerns about research ethics. There are two changes that I think are required before the paper is suitable for publication: (1) further detail and potential changes to the analyses in relation to hook depth information, and (2) consideration of bycatch rates predicted from only the significant predictors in the GAMM. My detailed comments are provided in the attached file.**

**One idea that the authors could add to the Introduction is that the landings ban for mako in the North Atlantic was also intended to change fishing behaviour. The reasoning was that mako captures would cause losses (time, gear, etc.) and no monetary gain, providing an incentive to avoid interactions in the first place.**

We revised the sentence on lines 121 – 123 to include a reference to changing fisher behavior. Revised sentence reads: “Retention bans aim to reduce fishing mortality to allow for population recovery by removing direct mortality associated with retaining individuals, and potentially by altering fisher behavior to avoid capturing the species.”

**When quantifying the differences in fishing characteristics between the GOM and WNA, I was particularly interested in the information on hook depth. Where does this information come from? The discussion implies that hook depth information comes from POP observers, so is it possible that the relatively small differences (10s of m) reflect different estimation ability of observers in the two regions? Line 245 suggests that at least one longline set had hook depths of 2m (the lower boundary of the range), which seems improbable given the catenary curve of a mainline between floats combined with gangion lengths. Table 2 lists ‘approximate’ hook depths, yet values are presented as known elsewhere in the MS, so does this mean there are two sources? Throughout the MS, please identify and discuss how hook depth was determined and (more importantly) quantify underlying variability if the values were estimated. This would help the reader to assess if the weak relationships with hook depth from the regression analyses are meaningful, particularly given that they take different shapes among regions.**

Yes, the hook depths are estimated by the observers and represent the best estimate of maximum hook depth. We better define what hook depth means in this study and describe how the depth is estimated on lines 384 – 386.

We address that a single estimate is unlikely to be fully representative of every hook in a set and that hook depth likely varies for the reasons the reviewer listed, and how that may have reduced our ability to detect a significant effect of depth on AVS in the discussion on lines 1230 -1234.

**From my understanding of the MC simulation, the predicted relationships for all variables were sampled, even those that were non-significant and/or had very little evidence of a relationship. I am concerned that this approach exaggerates the differences in bycatch survival among the two regions. I would like to see an analysis using only the significant predictors from each region in the MC simulation and present the analysis now in the MS as a sensitivity analysis. If the authors have supporting literature demonstrating that their current approach is more statistically rigorous, this should be added as justification.**

In the revised analysis, all covariates used in the AVS model in the MC simulation were meaningful (significant).

**Specific Comments:**

**Line 66: Potential addition: ‘….. and that overfishing is occurring with high probability [8]’.**

Done.

**Line 71: perhaps ‘fully implemented’ is better terminology than ‘adopted’, given that there is always a time lag between when ICCAT recommendations are adopted and when they are fully in force in various jurisdictions**.

Revised as suggested.

**Line 145-150: It would be worth mentioning why the modeling was split into two components, first a GLMM and then region-specific GAMMs. Presumably because this allowed for different relationships with predictor variables among regions?**

In the revised analysis we rely entirely on GLMMs.

**Line 145-150: Were any interactions tested in the suite of models? After reading the results, I was interested if there was any evidence for an interaction between SST and soak time for WNA. This would also bolster the information in the discussion (lines 402-416).**

In our revised AVS model we did consider interactions in our candidate models, mentioned on lines 418-420, and all candidate models are presented in Table 4.

**Line 160: Is 0.7 a common threshold for collinearity? Please provide a supporting reference.**

Yes, it is. We included the reference:

Dormann CF, Elith J, Bacher S, Buchmann C, Carl G, Carré G, García Marquéz JR, Gruber B, Lafourcade B, Leitão PJ, et al. (2013) Collinearity: a review of methods to deal with it and a simulation study evaluating their performance. Ecography 36(1): 27–46

**Line 187: Does the US fleet use wire leaders with weights or monofilament?**

Monofilament, at least in the Atlantic. We revised to “monofilament leader” here (line 505).

**Line 217: Section on Bycatch survival. It would be helpful to a reader to have a specific statement on why the simulation was done. This would highlight how your analysis better captures uncertainty in both estimated rates when generating the overall value, as compared to other analyses that merely multiply the individual means**.

Good idea. We added text explaining this on lines 569 – 593.

**Line 251: Were records considered incomplete if a single covariate was missing?**

For the analysis examining the influence of covariates on AVS we only included records where all covariates were available. We clarify this on lines 415 – 418.

**Line 265: ‘…. holding other variables constant at their predicted mean, …..’ Similar comment for Line 302.**

This specific line does not exist in the revised manuscript, but we used the suggested language consistently in the revised results on lines 782 – 789.

**Line 353: consider replacing ‘=’ with ‘of’**.

Revised as suggested.

**Lines 355-357 Re: bycatch survival. It would help the reader to have a simple statement that these are overall estimates (AVS*PRS).**

Revised to “Monte Carlo simulations combining our estimates of AVS and PRS suggested…” (line 919).

**Line 418: Figure 1 doesn’t show the depth ranges that sharks were hooked at in the NWA or GOM. Maybe reference Figure 2?**

Good catch. We checked to make sure we reference figures appropriately in the revised manuscript

**Line 417-418: For an animal that regularly dives from the surface to > 400 m in a day (Figure 5), I have a hard time with the idea that the difference in mean hook depth between the two regions is biologically meaningful. Typically shallow-set longline is differentiated from deep-set longline by 100s not 10s of meters, with the associated expectation of differences in AVS and PRS**.

In the revised analysis depth was not found to be informative. Regional variation in hook depth is presented in figure 2 of the revised manuscript.

**Line 536-537: Please reword to ‘…. as possible are associated with very high survival probabilities.’ Without assessing covariate effects, it is not possible to say that these actions maximize survival probabilities.**

Revised as suggested – line 1415.

**Figure 2: There seem to be no values on the x axes on each panel?**

This figure does not exist in the revised manuscript. But we were sure to include appropriate values for all axes.

**Supplementary File S2. Has the data been cleaned to only represent diving behaviour? Or are there still the portions of the tracks that represent times when the tag was at constant depth and/or drifting at the surface? Please clarify.**

Revised to indicate it is the full track.

**Reviewer #2: This study presents the results of an important data collection on the survival of mako sharks caught by commercial pelagic longline fisheries (US) in the Northwest Atlantic. In this study, the authors assess the survival (i.e. at-vessel survival AVS and post-release survival PRS) of the mako shark as a bycatch specie in the LL fisheries and identify the effects of environmental, biological and fishing practices variables on at-vessel survival. Given their results, the authors discuss the retention ban as a bycatch mitigation measure to the restoration of the abundance level of overfished population.**

**The manuscript is interesting because it provides important and much-needed data on total mortality (at-vessel and post-release survival) in a species that is highly vulnerable to pelagic longline fisheries, providing a more complete analysis of the impact of this fishery on mako sharks. It also tackles the issue of pelagic sharks’ fishing mortality in a new way through a study focusing on a comparison between two adjacent fishing regions of the north-western Atlantic.**

**However, the manuscript lacks scientific rigor at several levels:
The manuscript requires improvement in its use of standard English and scientific writing. Some sentences are not complete. (See my detailed comments below for specific examples).**
**General connections and shortcuts are made between different notions of fisheries, without the underlying logical links being clearly explained (e.g. stock assessment, risk assessment, overall mortality, retention ban...).**

It is unclear what the reviewer is referring to here. As such, it is hard to make any edits. We did remove the term “risk assessment” from line 955.

**The manuscript lacks consistency in the use of the terms “mortality” and “survival”. I think the authors should choose one or the other, but not both as it becomes confusing to follow the logical reasoning**.
We understand how this could be confusing. There is variability in what is reported (survival or mortality) in the literature that is cited. To help ameliorate this issue, starting on line 129, after introducing and defining AVM and PRM, we define AVS and PRS, then refer to survival for the rest of the manuscript. Note that when referring to other literature, this sometimes required us to calculate survival when a cited study only presented mortality. It is an easy enough fix, as survival = 1 – mortality.

**There is a lack of information on the characteristics of the fishing set observed, in particular their total number, and on gear configuration: the types of hooks used (and in what proportions), the type of bait used, the length of branchlines...
Also, the target species drives fishing practices (day or night) and gear configuration (especially hook depth and bait). This is a very important aspect to consider in this type of study. However, this information is not given (especially the proportions) in the data collected, and above all is not addressed in the discussion.**
We included a table (Table 1) that shows, for each of the regions with > 100 mako shark observations, the target species of observed sets, mean and range of hook depth values observed for each target species, the proportion of the total sets observed for each target species, the proportion of recorded mako shark catches in each region by target species. We summarize this information, along with providing the total number of sets observed, in the first paragraph of the results (lines 597-605).

We included a new figure (Figure 2) which shows the regional distributions (in the form of boxplots) of SST, hook depth, soak time, mainline length, and leader length for observed sets in which mako sharks were captured. We summarize regional differences in the text on lines 627-641.

We did not have data on hook type, although circle hooks have been required in the US PLL fleet since 2004 (explained on lines: 404-405), thus it’s likely only a very small proportion of the sharks in this dataset were captured on J-hooks.

We did not include bait type or time of day in our analyses because although these variables may influence catchability, they are unlikely to directly influence AVS.

**There is a lack of information on data processing, especially concerning hook depths. What do they correspond to and how were they calculated? The same goes for soak time: is it the soak time of the longline or the soak time of the mainline section between wo floats on which the shark was caught?**
The hook depths are estimated by the observers and represent the best estimate of maximum hook depth. We better define what hook depth means in this study and describe how the depth is estimated on lines 384 – 386.

Observers only record a single value of soak time for a set. It is for the entire line, encompassing the time between when the gear was set until with was retrieved. We describe this on lines 372 – 374.

**The hypothesis behind the modeling of the effect of each variable on mortality must be indicated and referenced**.
The rationale and hypothesized effects for each variable considered are presented on lines 319 – 392.

**Although this is an original and interesting approach carried out with an impressive dataset, it is not clearly explained why the authors compare these two different regions in terms of mako sharks AVS. Also, it is unclear why the authors chose the longitude as the sole criterion to discriminate the two regions**.
We did not choose longitude as the sole criterion. As described in lines 92 – 103 of the original manuscript, we choose those regions because they are ecologically distinct, are the areas with the greatest observations of mako shark captures and they have different fishing practices.

In the revised manuscript we did not create separate regional models to determine the influence of covariates on AVS. But as variation in survival is an important aspect of this study, we revised that paragraph in the introduction a bit to point out that mako sharks are captured over a large swath of the western Atlantic, that environmental conditions vary widely across such a large area, that fishing practices and target species will vary spatially as well because fishers respond to the different environmental conditions, and that the regional variations in fishing practices and environmental conditions is likely to lead to differences in bycatch survival (lines 145 – 204).

**I suggest the authors include the variable “sex of the animal” in the analysis. I'd advise performing an analysis on the reduced data matrix to assess whether “Sex” could have an effect on AVS - which could have management consequences, especially for populations where individuals of different sex may display different spatial distributions.**
Only including observations where sex was recorded and all AVS covariates of interest were recorded would have severely reduced sample size and statistical power. But the reviewer makes a good point that it is plausible for AVS to vary between sexes.

To address this, we first tested whether there was evidence of a difference in AVS between the sexes. Finding no significant effect of sex, we then were able to use all observations in which covariates of interest were available, regardless of if sex was recorded, to maximize sample size and statistical power when trying to determine which variables influenced AVS.

This is detailed in the methods on lines 411-418. The results are presented in Table 3 and on lines 654-656.

**As GAMM results show linear relationships in almost all covariates, I would suggest using GLMMs, which would also allow them to know the weight of each covariate on the variability of the dataset. Rerunning the models by selecting only those covariates with a significant effect will increase the level of predictability of the model, which as presented in the study, is rather poor. A careful interpretation of AUC results is needed to present the significance of the selected model.**
We completely revised the AVS analysis. Rather than use two GAMMS to determine influence of covariates on AVS for two regions independently, we used GLMMs as suggested and included all observations where covariate data was available for the entire POP dataset in a single model. Switching to GLMMs allowed us to directly compare the effect sizes of covariates. (especially considering we scaled and centered variables before model fitting). It also allowed us to construct and test 35 different candidate models and assess their relative support using AIC. In the end the top supported model, which was used to make inferences and to estimate bycatch survival only included informative covariates.

This is described in the methods on lines 410- 423 and lines 459 – 473 (AIC model selection).

Model selection results are presented in the text on lines 656 – 662, and full candidate model set with relevant AIC metrics is provided in Table 4.

The fixed-effects regression coefficients are presented in Figure 3, and visual plots of the predicted effects of the 4 informative covariates are presented in Figure 4. Informative parameters are presented in the text of the results on lines 782 – 789.

The methods for the leave-one-out cross validation applied to the top supported model are on lines 466 – 473.

The AUC result (0.58) is presented on line 663.

**l.59: The authors should define fishing mortality (at-vessel mortality + catch dying on board ? + post-release mortality ?)**
We use the standard definition of fishing mortality (F), encompassing all mortalities directly linked to the fishing process. We stated this in the text on lines 126 – 127.

**l.66-68: “Additionally, the International Union for the Conservation of Nature (IUCN) assessment estimated a ~60% decline in biomass over 75 years [9].” à the authors sould repeat which biomass they are talking about.**
Revised as suggested.

**l.72: concerning retention ban efficiency, authors should cite Tolotti et al., 2015: “Banning is not enough: The complexities of oceanic shark management by tuna regional fisheries management organizations**”
We added this citation, line 128.

**l.75-77 : the authors state something in the introduction that they are trying to show through their study**

We do not understand this comment.

**l.94-96 : missing reference**

Added citation to text about regional environmental differences (line 199).

**l.98-103: As I said in my general comments, actually it mainly depends on the target species, for which the depth of capture is adapted. I think it's worth giving other examples of differences in practices that can influence mortality than just depth, such as the type of hook, the type of line, the bait.... There are many references on this subject.**
This paragraph was considerably revised as described above. Examples of factors that could influence survival are provided on lines 142 – 144.

**l.105-112: Fig.2: it would be preferable to indicate the CPUE of mako and/or the proportion of mako observed in the total catch rather than just the number of catches, which may conceal very different fishing and/or observation efforts.**
**l.134-137: why are there so many differences between regions? is it possible to have the respective fishing efforts in each region?**

We added CPUE to Figure 1.

Effort data isn’t readily extractible from the POP data set, but the regional differences are likely a combination of effort and the distribution of mako sharks – the areas with the highest numbers correspond to the regions most used by makos based on fishery-independent tagging studies.

**l.137-139: the authors need to justify better than “for analysis purposes”, because these two regions don't have the same observation efforts, the same surface areas covered, and so the environmental variables are surely more variable for WNA than GOM.**
**It would be interesting to test the distribution of the covariates within the WNA zone to find out how homogeneous it is before grouping these regions together and assuming that the effects aez constant within the region.**

This comment is no longer relevant, as we analyzed all regions in a single model.

However, the distribution of covariates between regions with > 100 mako observations are presented in Figure 2.

**l.147: The choice of considering "year" as a random effect is unclear to me in this context. If information on vessel ID is available, it would be more appropriate to retain this covariate, as studies have suggested that variability in survival occurs at the fishing unit level (which summarizes a set of drivers specific to a captain’s fishing practices)**
We expand on our rationale for use of year as a random effect on lines 395-405, but it breaks down to accounting for random annual variation, differences in observer coverage, and changes in things such as regulations that would not otherwise be directly measured in the data available to us. Using individual set as a random effect would be ideal as the covariates were collected at the level of the set, but as most sets only contained a single shark such models would not converge, and we communicate this on line 405-408. We did not have access to vessel ID, which we communicate on lines 408-409.

**l.226-228: why these limits? references? or is it the distribution tail of their data? what thresholds have been chosen?**
The thresholds are based on lower limits observed in the actual data, and shark size was based on expected size of a new-born mako shark. We added explanations on lines 557-561.

**l.246-255 : switch between at-vessel mortality et survival**
The line referenced is no longer in the revised manuscript. But per previous reply, we made consistent reference to survival.

**l.263-264 : “although the effect of neither variable was very strong ». What is a strong effect ? table 1: I suggest that the authors indicate the coefficients, at least to know whether the effect is positive or negative.**
We did not report coefficients in the original manuscript because GAMMs do not output regression coefficients. With the revised GLMM approach, we report the regression coefficients in Figure 3.

**l.317 & 319: pay attention to meaning of the sentence, sharks don’t transmit data**
Revised this sentence “…27 tags deployed on sharks ranging from 91 – 270 cm FL transmitted data”

**l.342-343: The authors should indicate the type of hook that have been used in the methods part, and in what proportions. The hooking location is essential for survival. It would be nice to show this data for all sharks of the study, not only those that have been tagged, if the data is available of course. Similarly, whether or not sharks leave with the hook is vital information, which, if collected, should be intergrated as a potential mortality factor.**
We agree it would be great to having hook location for all sharks, but we did not have that data for the large historical POP dataset. We were able to ask for that data to be collected for the tagged sharks, but it’s not normally collected.

**table2: “approximate hook depth”: as indicated above: what does this hook depth correspond to? How was it calculated and at what point in the capture does it correspond?**
The hook depths are estimated by the observers and represent the best estimate of maximum hook depth. We better define what hook depth means in this study and describe how the depth is estimated on lines 384 – 386.

**l.375 : replace “anonymous” with “ICCAT**”
We think the reviewer has the request backwards? The original text says ICCAT. We revised to “Recent projections suggested that even…” and just placed the citation at the end of the sentence.

**l.376: “the north Atlantic stock is expected to continue to decline ... ” once again, pay attention to the sentence structure: you need to repeat which species you're talking about.**
Revised as suggested.

**l.377: I suggest that the authors indicate the quantities of mako fished currently and/or before the retention ban, in relation to the 300T TAC. Otherwise it is difficult to appreciate the seriousness of the current situation.**
We report catches reported from 2015 – 2017 as that is the available published data, lines 967-968.

**l.381-385: if the authors address the northern Atlantic mako stock, then they have to refer to the landing figures of the other fleets that fish this stock. Also, the last sentence suggests that the retention ban could be a problem, but I'm not sure that's what the authors meant.**
Proportion of landings attributed to the countries with the greatest proportion of landings is included on lines 974-975.

We revised this sentence to hopefully clarify our meaning – that low bycatch survival could slow population recovery in the presence of a retention band IF large numbers of makos are captured as bycatch (lines 972-1029).

**l.394: how do the authors interpret these similarities with the results of the portuguese fleet?**
We aren’t confident we have enough information to develop an explanation given the informational available to us that wouldn’t be overly speculative. As such we refrain from speculating on this.

**l.396-398: if this is the authors’ hypothesis, they can verify it with the data by running the models on the whole dataset, without discriminating the two regions.**
This comment is no longer relevant as we did revise the analysis to include the whole Atlantic. Still, we did find significant covariate effects on AVS. We offer a potential explanation for this difference with Gallagher et al on lines 1042-1044. Ultimately, we suspect that the shorter time period and data filtering they used may have reduced their power to detect effects.

**l.402: I suggest that the authors link the effect of temperature to that of hook depth. The subject is touched in lines 412 to 414, but not sufficiently developed. The effect of SST on the survival of animals caught at different depths and temperatures is therefore difficult to interpret, unless all individuals were caught above the thermocline, but this would have to be verified with data on hooking depths and thermocline to know.**
Much of this comment is not directly relevant given the revised analysis and its results. We did find temperature to be a significant driver of AVS in the revised analysis. In the paragraph on lines 1045 -1108, we discuss this finding as offering a potential solution to why AVS decreased in a north-south orientation, as warmer temperature in southern regions may confer a lower baseline mortality than in the cooler northern regions.

On lines 1227-1230 we communicate how although SST was the measure we had available, direct measure of temperature at hook depth might increase model performance and is something worth exploring in future studies.

**l.403-404: Given the method used, I don't find it justified to compare the results of driver effects between regions.. The authors looked at the effect of several variables for each region, not the effect of each variable between the two regions**.
Comment no longer relevant given the revised AVS analysis.

**l.408-409: The effect of SST on survival concerns sharks caught in the WNA, so this result should not be compared with those caught in the GOM. This interpretation would have been correct if the effect had been found for the whole sample.**
See reply to comment on line 402.

**l.424-427: I think that there are many explanations behind the fishing practices related to target species. This was not mentioned in the introduction or the methods, but it is important to indicate the proportions of fishing sets that targeted this or that species, and perhaps further analysis could integrate the target species as a driver of AVS.**
We included a table (Table 1) that shows, for each of the regions with > 100 mako shark observations, the target species of observed sets, mean and range of hook depth values observed for each target species, the proportion of the total sets observed for each target species, the proportion of recorded mako shark catches in each region by target species. We summarize this information in the first paragraph of the results (lines 597 – 607).

**l.463-464: just because the PRS is higher than the AVS does not mean that mako have a better chance of survival after release. I don't find it relevant to compare the two indicators. Otherwise in this case, the authors could compare with other species for which there would not be the same pattern.**
We revised this sentence to say “We found mako shark PRS (0.87) was relatively high, suggesting mako sharks released alive have a high probability of surviving.” Line 1300-1301.

**l.529: It would be relevant to place bycatch reduction in the context of a sequential mitigation hierarchy of interactions between fishing gear and the non-target resource (Gilman et al., 2023 - Bycatch-neutral fisheries through a sequential mitigation hierarchy - Marine Policy).**

We incorporated this suggestion (line 1417-1418).

**Reviewer #3: This is an interesting manuscript that presents an analysis of the survival of mako sharks (Isurus oxyrinchus), based on long-term observations from the Pelagic Observation Programme and telemetry data. The survival of mako sharks caught on longlines remains poorly understood. These sharks often experience high levels of stress and physical injury during capture, which can result in significant mortality. Their high activity levels and aggressive behavior may further increase the risk of injury. Using long-term data and advanced biotelemetry techniques, the authors analyze the survival of this species in two regions. They also propose conditions that could be maintained to improve the survival of mako sharks in longline fisheries.

The authors used data from 6681 observations made in the Western North Atlantic (WNA) and the Gulf of Mexico (GOM). The spatial distribution of the data was not uniform, so the authors correctly divided the material into GOM and WNA. The second source of data was the results of telemetry surveys using pop-up archival tags. This type of tag transmits via satellite the parameters recorded over a pre-determined period of time. In this case, the material was only from the years 2022-2024. The data to determine post-release survival were collected over a limited area. However, the authors selected the area with the highest number of mako shark sightings for this element of the study. Based on the depth and temperature recorded, the authors estimated the medium-term mortality of this species. In conclusion, I believe that the authors have prepared an interesting manuscript, used appropriate data analysis methods and presented the results clearly. In the discussion chapter, I like the caution in making explicit assessments. The authors are aware of the weaknesses of their study. They rightly consider that the soaking time was not the best indicator of the time spent on the line. They also address the smaller sample size in the GOM, stressing that it may not have been sufficient to detect the effect.
Before accepting this manuscript for publication, I suggest that you consider some of the following points.**
We appreciate the positive words!

**1. Surprisingly, SST was only a significant factor in increased mako mortality in the colder waters of the WNA. I lack an explanation as to why this factor did not have a significant effect on AVS in the warmer GOM? Or is this an adaptation to local conditions?**
In the revised analysis, we analyzed all the data together. We still found a significant effect of SST. In the original analysis we suspect the reason there was no effect detected in the GOM was that there was little variation in the region (temperatures were universally warm) whereas sharks were caught in a wide range of temperatures in the NWA, which allowed for the effect to be detected.

**2. Why were there regional differences in AVS for mako sharks? What could have been the reasons for the significant differences in the GOM and WNA? The importance of immersion time is particularly surprising. Was this due to differences in depth or some other reason? Does the soak time depend on the depth of the fishery or some other technicality?**
While the revised analysis did not compare the same regions, we did still find significant regional variation between the northern observation regions (MAB, NEC, NED) and the southern observation regions (SAB, FEC, GOM). We suggest that water temperature may be a driving force for these differences, as of the significant covariates it was the one that varied most between regions. We explain this in the paragraph on lines 1045-1108 in the discussion.

**3. The case of the mortality of a mako shark on day 14 after release is very interesting. What endothermic predator could have attacked/eaten such a large shark? Could this predator have a temperature of 22.5-26.9 degrees Celsius? What is the accuracy of the temperature readings from the pop-up tags used?**
Based on the temperatures and the region of the ocean it was likely a larger lamnid shark; either a white shark or a larger mako. We added text about this on lines 809-887.
We added the temperature and depth accuracy of the tags on line 481.

**Specific comments:
Lines 131-133: Reference to more recent literature is needed. Publication 29 (Diaz et al. 2009) only gives data up to 2007. Where does it say that after 2007 observer coverage was >8%?**
We added the range of actual coverage available in annual reports for the years 2007 – 2019 on lines 310-312. The range was 10 – 16% each year.

**Line 142: What do you mean by hook depth? For those unfamiliar with this fishing technique, an explanation of the term is needed.**
The hook depths are estimated by the observers and represent the best estimate of maximum hook depth. We better define what hook depth means in this study and describe how the depth is estimated on lines 384 – 386.

**Line 187: How was the leader length estimated? Who did it? What was the margin of error? Was this person experienced in this procedure?**
Leader lengths were estimated by fisheries observer, estimated to the nearest inch. Added on line 505-506.

**Lines 347-350: why did you not describe this analytical procedure in the methods section?**
We moved the description of the fishers exact test procedure to the methods section (lines 532-534).

**Lines 381-382: Where did you get the data on the US contribution to total landings? References are needed.**
We added a reference for this information

National Marine Fisheries Service (NMFS). (2018) Final Environmental Assessment for Emergency Interim Final Rule to Address Overfishing of North Atlantic Shortfin Mako Shark. Highly Migratory Species Management Division, Office of Sustainable Fisheries, NOAA Fisheries, Silver Spring, MD.

**Line 478: Information that all PLL fleets used circle hooks should be in the methods section. Information on the type of hook used to catch fish is important when interpreting the data in the context of survival. This is the only place where such information appears, and readers with knowledge of the subject will find it easier to follow the authors' reasoning.**
We added information on circle hook use in the methods on lines 404-405.

**Figures - their quality is poor and should be improved. I think that higher dpi values should be used.**

We uploaded higher resolution figures.
